# Selecting costimulatory domains for chimeric antigen receptors: functional and clinical considerations

**DOI:** 10.1002/cti2.1049

**Published:** 2019-05-11

**Authors:** Robert Weinkove, Philip George, Nathaniel Dasyam, Alexander D McLellan

**Affiliations:** ^1^ Cancer Immunotherapy Programme Malaghan Institute of Medical Research Wellington New Zealand; ^2^ Wellington Blood & Cancer Centre Capital & Coast District Health Board Wellington New Zealand; ^3^ Department of Pathology & Molecular Medicine University of Otago Wellington Wellington New Zealand; ^4^ Department of Microbiology and Immunology University of Otago Dunedin New Zealand

**Keywords:** immunology, immunotherapy, lymphocytes, T cells

## Abstract

Costimulatory signals are required to achieve robust chimeric antigen receptor (CAR) T cell expansion, function, persistence and antitumor activity. These can be provided by incorporating intracellular signalling domains from one or more T cell costimulatory molecules, such as CD28 or 4‐1BB, into the CAR. The selection and positioning of costimulatory domains within a CAR construct influence CAR T cell function and fate, and clinical experience of autologous anti‐CD19 CAR T cell therapies suggests that costimulatory domains have differential impacts on CAR T cell kinetics, cytotoxic function and potentially safety profile. The clinical impacts of combining costimulatory domains and of alternative costimulatory domains are not yet clearly established, and may be construct‐ and disease‐specific. The aim of this review is to summarise the function and effect of established and emerging costimulatory domains and their combinations within CAR T cells.

## T cell costimulation

Antigen‐specific activation of T lymphocytes (T cells) relies on interactions between T cell receptors (TCRs) and their cognate antigens, which lead to TCR aggregation, phosphorylation of TCR‐associated CD3 proteins and downstream intracellular signalling.^1^ However, the strength and quality of a T cell response to antigen is highly dependent on signalling by an array of costimulatory and co‐inhibitory receptors also expressed on the T cell surface.^2^ Costimulatory signals enhance T cell proliferation, cytokine secretion, cytotoxic function, memory formation or survival. In contrast, co‐inhibitory signals inhibit T cell proliferation and function and can lead to tolerance, exhaustion and T cell apoptosis.^1^

As early as 1975, Lafferty and Cunningham^2^ proposed that in addition to antigenic stimuli, full activation of antigen‐specific T cells required secondary signals from antigen‐presenting cells (APCs). Confirmation of this classical two‐signal hypothesis was achieved with the generation of antibodies that block costimulatory molecules, and the cloning of B7 family members CD80 and CD86, which are ligands for the prototypical T cell costimulatory receptor CD28.^3^ Later observations confirmed that antigenic stimulation without sufficient costimulation results in T cell anergy and T cell susceptibility to activation‐induced cell death (AICD).^3^

Broadly defined as cell surface molecules that can transduce signals into T cells to enhance TCR‐mediated signals,^1^ most costimulatory receptors belong to the immunoglobulin (Ig) or tumor necrosis factor receptor (TNFR) superfamilies, and bind to ligands expressed by activated or licensed APCs.^3^, ^4^ Costimulatory receptors of the Ig superfamily tend to activate phosphatidylinositol 3‐kinase (PI3K) leading to protein kinase B (Akt) and downstream nuclear factor κB (NF‐κB) activation, while TNFR superfamily members preferentially recruit and activate TNF receptor‐associated factors (TRAFs) to potentiate NF‐κB activation.^3^, ^4^

Examples of costimulatory molecules expressed by T cells include CD28, ICOS, CD27, 4‐1BB, OX40 and CD40L (see Table 1). The degree of costimulation received by a T cell is modulated by T cell surface expression of costimulatory molecules, expression of their ligands on APCs and T cell expression of co‐inhibitory receptors, many of which compete with costimulatory receptors for the same ligands. In physiological and infectious disease settings, costimulatory signals are delivered by APCs in response to danger signals, such as pathogen‐associated molecular patterns, or stressed or necrotic cells, or in response to ‘licensing’ of peptide‐presenting APCs by CD4^+^ T‐helper cells.^1^, ^3^ Mice lacking key costimulatory receptors, such as CD28 or 4‐1BB, exhibit defects in T cell function.^3^, ^5^ In contrast, monoclonal antibodies that either activate T cell costimulatory receptors or block co‐inhibitory receptor signalling can enhance T cell responses against cancer.^3^, ^6^ However, activation of costimulatory receptors can lead to unbridled inflammatory responses with life‐threatening consequences.^7^ An understanding of T cell costimulation is therefore critical for the development of safe and effective adoptive T cell therapies, including those employing chimeric antigen receptors CARs.

**Table 1:** Costimulatory molecules employed within chimeric antigen receptor (CARs)

Costimulatory molecule	Ligand(s)	T cell expression	Functional characteristic(s) within CAR T cell
Ig superfamily			
CD28	CD80/CD86	Resting and activated T cells	Potent cytotoxic function; IL‐2 production; may favor CD4^+^ T cell expansion
ICOS (CD278)	ICOS‐L (CD275)	Activated T cells, especially Tfh and Th17 cells	May favor Th1 and Th17 polarisation
Tumor necrosis factor receptor superfamily			
4‐1BB (CD137)	4‐1BBL (CD137L)	Memory and CD8^+^ T cells; CD4^+^ T cells only upon activation	Stimulates CD8^+^ central memory T cell generation. Favors CAR T cell persistence
OX40 (CD134)	OX40L (CD252)	Activated T cells	Suppresses Treg development
CD27	CD70	Activated T cells	Upregulated Bcl‐X(L) protein expression. Favors CAR T cell persistence
CD40	CD40L (CD154)	Activated T cells	Increases proliferation and secretion of pro‐inflammatory Th1 cytokines
Others			
CD40L	CD40	Activated T cells	Acts indirectly on tumor cells or antigen‐presenting cell through the enhancement of costimulatory activity and cytokine release
TLRs	TLR agonists	Activated T cells	Enhances effector function and cytotoxicity; increases IL‐2, IFN‐γ and GM‐CSF production

## Costimulatory domains within chimeric antigen receptors

Synthetic ‘CARs’ represent a major advance in adoptive T cell therapy. By combining the variable regions of a high‐affinity monoclonal antibody with intracellular signalling components derived from the TCR complex, CARs allow redirection of T cell cytotoxicity against an antigen of choice, entirely independently of target cell major histocompatibility complex (MHC) expression.

For discussion purposes, in this section, the first domain (including the transmembrane (TM), domain) listed is that closest to the membrane, and the last is at the cytoplasmic tail. Initial CAR designs incorporated the variable regions of a monoclonal antibody fused to the TCR α‐ and β‐chains.^8^, ^9^ Subsequently, modified versions utilised an ‘ectodomain’ of single‐chain variable fragment (scFv), a transmembrane domain and an ‘endodomain’ incorporating immunoreceptor tyrosine‐based activation motif (ITAM)‐replete FcγR or CD3‐ζ domains.^10^ The scFv is typically of high affinity for the corresponding antigen and contributes to observed differences in signalling between conventional TCRs and CAR constructs. This basic structure has formed the basis for modern CAR design.

**Figure 1:**
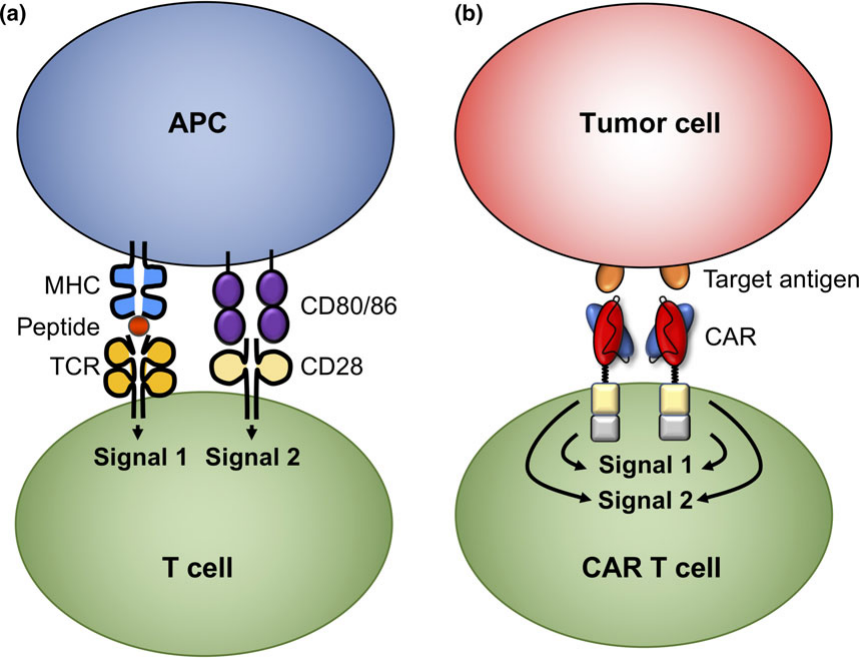
Costimulation in unmodified T cells and within chimeric antigen receptor (CAR) T cells. **(a)** T cells express a TCR specific for peptide in the context of major histocompatibility complex. Costimulation eliciting optimal T cell activity, proliferation and survival requires expression of costimulatory receptor ligands such as CD80 and CD86 on APCs. **(b)** T cells transduced to present a CAR incorporating an intracellular costimulatory domain, such as that of CD28, can undergo potent activation upon exposure to cells expressing the target antigen without target cell expression of costimulatory receptor ligands. Since CAR costimulatory domains lack the original costimulator extracellular structures, they are unable to transmit negative signals from inhibitory ligands.

T cells genetically modified to express CARs incorporating intracellular CD3ζ alone, now termed ‘first‐generation’ CARs, are activated by and exhibit cytotoxicity against target cells expressing the CAR target, but failed to proliferate well or to elicit long‐term antitumor responses.^11^ Incorporation of the intracellular signalling domain of CD28 into a CAR improved T cell proliferation and cytokine production.^12^, ^13^ Figure 1 contrasts costimulation of unmodified T cells with CAR T cell costimulation. Incorporating a costimulatory receptor domain into the CAR itself, rather than within a separate transgene, or relying on costimulatory ligand expression by endogenous cells has distinct advantages: (1) a single polypeptide CAR incorporating only the intracellular signalling domain of the costimulatory molecule(s) is required, retaining a parsimonious transgene size, (2) costimulatory signalling is restricted to T cells both expressing the CAR and exposed to target antigen, and (3) costimulatory signals are provided independently of tumor or microenvironment expression of costimulatory receptor ligands.

**Figure 2:**
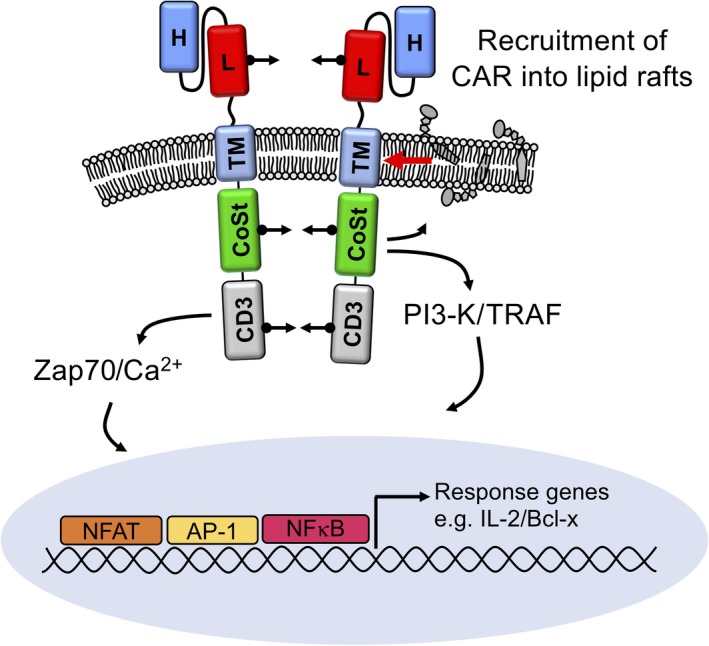
Generalised mechanism of chimeric antigen receptor (CAR) signalling provided by CD3‐ζ ITAMs and a costimulator domain. Ligation of the CAR scFv to tumor antigen provokes aggregation of the CAR polypeptide. CD3‐mediated activation proceeds via classical Zap70‐mediated pathway resulting in Ca^++^ influx and release from intracellular stores and translocation of de‐phosphorylated NFAT to the nucleus. Costimulatory domains preferentially recruit PI3 kinase and TRAF to enhance cytokine and cell survival gene transcription, particularly through AP‐1 and NF‐κB translocation to the nucleus. Costimulator domain signalling activates cytoskeletal mobilisation, enabling colocalisation of CAR to membrane rafts.

Following initial demonstrations that the CD28 domain within CARs was effective in providing costimulation, a range of other costimulatory domains have been assessed, including another Ig superfamily member, ICOS, and the TNFR superfamily members 4‐1BB, OX40 and CD27.^14^, ^15^ The use of costimulatory domains has since expanded from those derived from members of the Ig and TNFR superfamilies, to others signalling via cytoplasmic domains of IL‐2Rβ, IL‐15R‐α, CD40 or MyD88.^16^, ^17^ The incorporation of different costimulatory molecules into CARs may be expected to confer varying degrees of costimulatory domain‐specific activation, potentially with distinct impacts on CAR T cell activity, proliferation and fate (Figure 2).

### CD28

The Ig superfamily member CD28 is considered the prototypical T cell costimulatory receptor and competes with its co‐inhibitory receptor counterpart CTLA4 for binding to the B7 molecules CD80 and CD86 on APCs.^3^ While some costimulatory molecules are expressed only upon T cell activation, CD28 is expressed by both resting and recently activated T cells.^3^ The potency of unrestrained CD28 signalling is exemplified by the lethal lymphoproliferative syndrome occurring in CTLA4‐deficient mice, and by the severe inflammatory syndrome seen in clinical trial recipients of an agonistic anti‐CD28 monoclonal antibody (mAb) that binds to the membrane‐proximal loop of CD28.^3^ This CD28 superagonist can trigger profound T cell activation even in the absence of TCR ligation, overturning the long‐standing concept that two signals are always required for T cell activation.^2^, ^3^

Ligation of CD28 recruits the phosphatidylinositol 3‐kinase (PI3K), the adapter Grb‐2, the serine/threonine phosphatase PP2A and the protein tyrosine kinases Lck and Itk.^3^ The CD28 cytoplasmic motif YMNM binds and activates both PI3K and Grb‐2.^3^ This results in increased T cell proliferation and IL‐2 production by enhancing transcription and mRNA stability.^3^ CD28 signalling enhances resistance of T cells to AICD, an effect mediated by enhanced IL‐2 availability and the upregulation of pro‐survival pathways, and promotes production of Th1 cytokines such as IFN‐γ, TNF‐α and GM‐CSF. However, CD28 costimulation provided by synthetic CD3/CD28 microbeads or artificial APCs preferentially expands CD4^+^ T cells and may lead to exhaustion or anergy of CD8^+^ T cells.^18^

In addition to enabling T cell signal transduction, CD28 participates in reorganisation of the cytoskeleton.^3^ CD28 activates actin polymerisation via the action of guanine nucleotide exchange factor Vav1, together with activation of small Rho GTPase cell division control protein 42 (Cdc42), Wiskott–Aldrich syndrome protein and actin‐related protein 2/3 complex.^3^, ^19^ Actin polymerisation mediated by Vav1 and Filamin A has been proposed to recruit membrane rafts to the immune synapse and likely contributes to the observed enhancement of the tensile strength of the immune synapse following CD28 triggering.^19^ By stimulating the formation and migration of membrane rafts to the immune synapse, CD28 mobilises Lck, LAT and PKCθ to the TCR to further potentiate TCR signalling. Conversely, disruption of membrane raft formation impedes CD28 activation of the phosphoinositide 3‐kinase (PI3K)/Akt signalling pathway.^19^

Since CAR signalling domains are composed of fused CD3ζ ITAMs plus costimulatory motifs, signal transduction from second‐ and third‐generation CARs is a product of both costimulatory domain and CD3ζ signalling. The composition of both CD3ζ and costimulatory signalling domains on a single, high‐affinity, antigen receptor, and the replacement of positively charged amino acid TCR residues with uncharged residues on the TM of costimulatory molecules, may contribute to the atypical immune synapses observed in CAR T cells.^20^

*In vivo*, ‘second‐generation’ anti‐CD19 CARs using CD28 costimulation demonstrate improved antitumor efficacy and persistence in comparison with first‐generation CAR T cells lacking a costimulatory domain.^21^ Human CD8^**+**^ CD28‐costimulated CAR T cells have shown both central and effector memory phenotypes and exhibit rapid proliferation and IFN‐γ production *in vitro* upon recognition of target antigen.^22^, ^23^, ^24^, ^25^ Recruitment of regulatory T cells by CD28‐costimulated CAR T cell‐derived IL‐2 may limit antitumor activity in some models.^26^ However, in general, T cells expressing second‐generation CARs with CD28 costimulatory domains are associated with faster tumor elimination and activity at lower effector:target ratios compared to those expressing CARs with 4‐1BB domains.^27^

Consistent with animal studies, early‐phase clinical trials suggest that CD28 costimulation augments CAR T cell activity. Savoldo *et al*.^28^ simultaneously administered autologous anti‐CD19 CAR T cells both without (first‐generation) and with (second‐generation) an intracellular CD28 costimulatory domain to six patients with relapsed or refractory (r/r) B‐cell non‐Hodgkin's lymphoma (B‐NHL) without prior lymphodepleting chemotherapy. The CAR T cells lacking a CD28 costimulatory domain showed limited expansion and poor persistence, whereas the T cells expressing a CAR incorporating the CD28 domain underwent a greater degree of expansion and persisted for longer in all six patients.^28^

Autologous T cells expressing an anti‐CD19 CAR incorporating the intracellular CD28 costimulatory domain (axicabtagene ciloleucel) have been licensed for the treatment of r/r aggressive B‐NHL, are associated with very high response rates in the treatment of r/r B‐cell acute lymphoblastic leukaemia (B‐ALL) and have led to promising results in the treatment of chronic lymphocytic leukaemia and multiple myeloma.^11^, ^29^, ^30^

### 4‐1BB (CD137)

4‐1BB (*TNFRSF9*, CD137) is an activation‐induced T cell costimulatory molecule, first described in 1989.^6^ A TNFR superfamily member, 4‐1BB, is expressed on a subset of resting CD8^+^ T cells and is upregulated on both CD4^+^ and CD8^+^ T cells following activation.^31^ Upon binding to trimeric 4‐1BBL (*TNFSF9*, CD137L) on APCs, 4‐1BB recruits TNFR‐associated factor family members (TRAF1, TRAF2 and TRAF3) to its cytosolic region, forming the 4‐1BB signalosome and leading to downstream activation of NF‐κB, MAPK and ERK.^4^

Agonistic stimulation of 4‐1BB upregulates expression of the anti‐apoptotic proteins Bcl‐x_L_ and Bfl‐1 and prevents AICD,^6^ while 4‐1BB activation increases IL‐2 and IFN‐γ in CD8^+^ cells and IL‐2 and IL‐4 in CD4^+^ cells, the presence of two TRAF2 binding motifs being essential for downstream IL‐2 production.^6^^,^ 32 Although 4‐1BB costimulation enhances proliferation of both CD8^+^ and CD4^+^ T cells, it preferentially supports CD8^+^ T cell expansion, in contrast to CD28 costimulation.^31^ Potent antitumor cytotoxicity can be induced by the administration of agonistic 4‐1BB mAbs in several tumor models and in the clinic.^33^

T cells expressing CARs that incorporate 4‐1BB domains have been shown to express granzyme B, IFN‐γ, TNF‐α, GM‐CSF and the anti‐apoptotic protein Bcl‐x_L_.^22^ Incorporation of the 4‐1BB TM and cytoplasmic domain into a CAR leads to improved persistence and antitumor activity,^34^ and, compared to a CD28‐costimulated CAR, prolonged T cell division. However, CD28‐based CARs provide more rapid and profound alterations in protein phosphorylation and greater effector T cell activity.^35^ Kawalekar *et al*.^24^ reported that a CAR utilising a CD28 domain elicits an effector memory T cell phenotype with a gene signature suggesting glycolytic metabolism, while a 4‐1BB domain favors development of CD8^+^ central memory T cells with higher respiratory capacity and enhanced mitochondrial biogenesis. In clinical trials, second‐generation CARs incorporating a 4‐1BB costimulatory domain appear to favor longer CAR T cell persistence than those incorporating a CD28 domain.^27^, ^30^, ^36^

Different malignancies might benefit from different CAR T cell costimulatory domains: while the longer persistence of CAR T cells employing 4‐1BB costimulatory domains may be important for long‐term remission of the precursor B‐cell malignancy B‐ALL, long‐term CAR T cell persistence may be less critical than early antitumor activity when treating mature B‐NHL malignancies.^37^ However, variability in CAR design, in vector, in manufacturing processes and between patient cohorts precludes definitive comparisons across CD28 and 4‐1BB‐based CAR T cell clinical trials.

Autologous T cells expressing a second‐generation anti‐CD19 CAR incorporating the intracellular 4‐1BB costimulatory domain (tisagenlecleucel) have been licensed for the treatment of r/r B‐ALL and aggressive B‐NHL.^36^, ^38^

### ICOS (CD278)

Inducible T cell costimulator (*ICOS*, CD278), a CD28 family member, is upregulated upon T cell activation and binds to ICOS ligand (*ICOSLG,* CD275) on APCs.^1^ In an manner analogous to CD28, the binding of ICOS to its ligand activates the PI3K/Akt pathway within T cells.^1^ Both ICOS and CD28 recruit PI3K via a cytoplasmic YMXM motif (YMNM in CD28 and YMFM in ICOS), ICOS costimulation leading to greater PI3K activity than CD28.^39^ Unlike CD28, however, the TM domain of ICOS is sufficient to recruit the tyrosine kinase Lck and to augment TCR‐triggered calcium mobilisation.^40^ Furthermore, ICOS is unable to recruit Grb‐2, which may account for the reduced IL‐2 expression elicited by ICOS‐compared to CD28‐mediated costimulation.^41^

While CD28 is critical for the initiation of an immune response, ICOS costimulatory signals appear particularly important at the time of secondary antigen exposure.^42^ Costimulatory signals by ICOS promote T cell production of IL‐4, IL‐10 and IL‐21, and are considered critical for development and maintenance of Th2 and T follicular helper cells in mice,^1^ and of human Th17 cells.^43^

When expressed in human T cells, second‐generation CARs utilising an ICOS costimulatory domain lead to greater PI3K activation than those using 4‐1BB.^14^ Compared to CARs incorporating either CD28 or 4‐1BB costimulatory domains, ICOS‐based CARs lead to greater Th1/Th17 polarisation of T cells, with increased expression of IL‐17A, IL‐17F, IL‐22, IFN‐γ and T‐bet.^43^ In keeping with a particular role for ICOS within Th17 cells, human Th17 cells expressing second‐generation CARs with an ICOS costimulatory domain exhibit improved antitumor activity and persistence than those utilising a CD28 or 4‐1BB domain.^44^ Conversely, ICOS‐based CARs lead to lower levels of T cell IL‐2 production than do CD28‐based CARs.^43^

### OX40 (CD134)

The TNFR superfamily member OX40 (*TNFRSF4*, CD134) is expressed following T cell activation and, like 4‐1BB, is not involved in initial T cell activation, but is essential for T cell proliferation and survival.^45^ Upon binding to its ligand OX40L (*TNFSF4*, CD252) on activated APCs, OX40 acts via both PI3K/Akt and TRAF pathways. TRAF2, TRAF3 and TRAF5 associate with OX40 via a cytoplasmic QEE motif,^45^ TRAF3 mediating NF‐κB activation.^46^ OX40 enhances Bcl‐xL, but also upregulates Bcl‐2, Bfl‐1, survivin and aurora B kinase.^45^ Since OX40 lacks the YMXM PI3K binding site of CD28 and ICOS, it is not clear whether OX40 activates PI3K directly or indirectly.^45^ OX40 costimulation antagonises the activation and development of natural and inducible Tregs via suppression of CTLA4, TGF‐β and FoxP3 expression.^45^, ^47^

Addition of a OX40 endodomain to a second‐generation CD28‐costimulated CAR led to reduced CD28‐mediated IL‐10 secretion by CD4^+^ T cells, without altering expression of other pro‐inflammatory cytokines.^48^ In contrast, a study comparing a third‐generation CD28‐OX40‐ζ CAR to second‐generation CD28‐ζ or OX40‐ζ CARs found that incorporation of OX40 showed 10‐fold and fivefold enhancement in production of IL‐2 and IL‐10.^49^ Quintarelli *et al*. tested third‐generation CARs incorporating either OX40 or 4‐1BB alongside a CD28 costimulatory domain. They reported that constructs incorporating OX40 led to production of less INF‐γ and IL‐2 and exhibited reduced antitumor activity *in vivo*, compared with those incorporating 4‐1BB.^50^

### CD27

The TNFR family member CD27 (*TNFRSF7*) is constitutively expressed on CD4^+^ and CD8^+^ T cells and on a subset of NK cells.^46^ Unlike the other TNFR family members, CD27 is present at the cell surface as a disulphide‐linked homodimer and its expression is upregulated upon T cell activation.^46^ CD27 binds to CD70 (*TNFSF7,* CD27L), which is expressed by mature dendritic cells, activated B and T lymphocytes and some haematologic malignancies.

Incorporation of the CD27 cytoplasmic domain into a CAR endodomain resulted in CAR T cell upregulation of Bcl‐X(L) and enhanced resistance to apoptosis.^51^ The CD27‐based CAR demonstrated greater persistence than the CD28‐based CAR, similar to that observed with 4‐1BB CARs.^51^

### Other costimulatory domains

Costimulatory signals can be delivered to T cells by molecules outside the Ig and TNFR superfamilies. For example, a number of Toll‐like receptors (TLRs), as well as the TLR adaptor molecule MyD88, are expressed by activated T cells, and TLRs can serve as costimulatory molecules within T cells, augmenting T cell cytokine production and cytokine production in response to TCR stimulation.^52^, ^53^ TLR2 stimulation of human T cells, for example, leads to enhanced Akt and Erk1/Erk2 phosphorylation in the presence of TCR stimulation.^52^ The incorporation of a TLR2 endodomain into CD28‐costimulated second‐generation CARs enhanced CAR T cell activity against CD19 and mesothelin‐expressing tumors.^53^, ^54^

**Figure 3:**
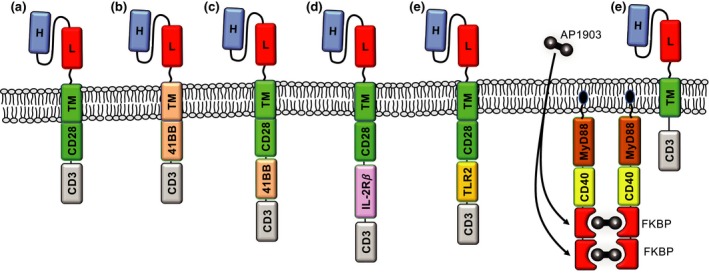
Examples of costimulator domain organisation within chimeric antigen receptor (CAR) constructs: **(a, b)** Second‐generation CAR, **(c–e)** third‐generation CAR and **(f)** an example of trans‐costimulation provided by small molecule‐mediated aggregation of the MyD88‐CD40 domains using AP1903.^17^ The inclusion of relevant transmembrane domains, as well as the orientation of the cytoplasmic domains, imparts critical functionality to the various CAR constructs.

A further innovation in accessory domains is the inclusion of cytoplasmic domains of common cytokine receptors into the CAR endodomain (Figure 3). Kagoya *et al*. engineered a truncated IL‐2Rβ cytoplasmic domain, together with a STAT3‐binding (YXXQ) motif into 4‐1BBζ and CD28ζ CARs. This modification enhanced CAR T cell proliferation and prevented terminal effector cell differentiation, and antitumor activity was reported to be superior to that elicited by CARs incorporating only CD28 or 4‐1BB costimulatory domains.^55^ IL‐15 is an effective driver of T cell proliferation, survival and memory cell formation. In a recent comparison of CD28‐based third‐generation CARs with the addition of 4‐1BB, CD27, OX40, ICOS or IL‐15Rα to the endodomain, Nair *et al*.^16^ reported that the cytoplasmic domain of IL‐15Rα resulted in the greatest expansion and the most rapid acquisition of effector cell function.

### Combining costimulatory domains

As noted earlier, CAR T cell activity could be enhanced by incorporating more than one costimulatory domain alongside CD3ζ, to produce a ‘third‐generation’ CAR (Figure 3).

*In vitro* and xenograft studies indicate that T cells expressing third‐generation CARs can combine the tumoricidal capacity of CD28‐based CARs with the persistence generated by 4‐1BB‐based CARs.^27^ For example, Zhao *et al*. found that expression of a third‐generation CAR construct combining a CD28 domain proximal to the membrane and a 4‐1BB domain distally leads to increased T cell expression of type 1 interferon pathway members, greater expansion of CD4^+^ and CD8^+^ T cells and improved B‐ALL tumor regression in xenografts, compared to second‐generation constructs. Lower doses of CAR T cells expressing the third‐generation construct were required for full antitumor activity, and the third‐generation CAR T cells displayed longer persistence than their second‐generation counterparts.^27^

Clinical data also indicate that third‐generation CARs lead to improved CAR T cell expansion and persistence. In a phase I dose–escalation study, Ramos *et al*.^56^ simultaneously administered third‐generation autologous anti‐CD19 CAR T cells (incorporating both CD28 and 4‐1BB costimulatory domains) and second‐generation CAR T cells (expressing only the CD28 costimulatory domain) to patients with r/r B‐NHL. Six of 11 patients with active disease responded to treatment, including three complete responses (CRs). One case of severe cytokine release syndrome (CRS) and one case of severe CAR T cell‐related encephalopathy syndrome (CRES), also referred to as immune effector cell‐associated neurotoxicity syndrome (ICANS), were observed.^56^, ^57^ In 10 out of 11 patients with active disease, greater expansion (up to 40‐fold) of third‐generation CAR T cells compared to second‐generation CAR T cells was seen, and third‐generation CAR T cells remained detectable at higher levels up to 160 days post‐infusion. Moreover, only third‐generation CAR T cells expanded significantly when infused to patients in remission after autologous stem cell transplantation, suggesting the third‐generation CAR T cells can expand despite minimal CD19 antigen exposure. Considering the results of Cheng *et al*.,^25^ who reported no significant difference between two‐second‐generation (CD28 vs 4‐1BB) CAR T cells co‐infused in patients, Ramos *et al*.^56^ concluded that third‐generation CAR T cell therapy may be effective in the eradication of minimal residual disease and lead to longer, more durable remissions.

Enblad *et al*.^58^ reported the outcomes of 15 patients with r/r B‐cell malignancies treated with autologous anti‐CD19 CAR T cells incorporating both CD28 and 4‐1BB costimulatory domains, four of whom did not receive lymphodepletion before CAR T cell infusion. Six achieved a CR, two of four with B‐ALL and four of 11 with B‐NHL. No treatment‐related mortality was reported, and three cases of severe CRS or neurotoxicity were observed, comparable to the toxicity rates observed with second‐generation CAR T cell therapies.^36^, ^58^, ^59^

Third‐generation CAR T cells incorporating the Toll/interleukin‐1 receptor (TIR) domain of Toll‐like receptor 2 (TLR2), together with the CD3ζ and CD28 intracellular domains, were administered to three patients with an extramedullary relapse of B‐ALL. All three patients achieved a CR; final results of a phase I dose–escalation study using this construct are awaited.^53^

Despite promising preclinical results and greater proliferative potential in early clinical trials, the clinical benefits of combining costimulatory domains within third‐generation CAR T cells are yet to be conclusively demonstrated. In particular, the optimal dose of third‐generation CAR T cell leading to improved clinical efficacy, without increased toxicity risk, is still unknown. A potential limitation of combining multiple costimulatory domains within a single CAR construct is that this might elicit tonic CAR signalling, leading to CAR T cell exhaustion, paradoxically reducing activity. If this proves a limitation, providing multiple populations of second‐generation CAR T cells, modifying second‐generation CAR T cells to express an additional full‐length costimulatory molecule in *trans* or providing inducible costimulatory signals might offer means to add costimulatory signals without inducing tonic signalling and exhaustion.^25^, ^27^

## Alternative mechanisms for the provision of costimulatory signals to CAR T cells

Incorporating the intracellular signalling domain of T cell costimulatory molecules into CARs is not the only means of providing costimulatory signals to CAR T cells. *In vivo,* CAR T cell costimulation may result from not only signalling via costimulatory molecule domains within the CAR, but also from full‐length costimulatory receptors expressed on the surface of the CAR T cells, expression of which may be enhanced by CAR T cell activation. Indeed, CD3/CD28 triggering, frequently used to activate T cells to enable lentiviral transduction during CAR T cell production, can upregulate the TNFR superfamily costimulatory molecules 4‐1BB (CD137) and OX40 (CD134), enhancing T cell persistence and memory.^60^, ^61^ While solid tumors might not be expected to express an abundance of costimulatory molecule ligands, malignant B cells can actively stimulate T cells via CD28, 4‐1BB, OX40 and CD40L.^62^, ^63^ This may explain in part the relative success of CAR T cells in treating B‐cell lymphomas, leukaemias and myeloma, compared to tumors of epithelial or mesenchymal cell origin. In addition, CAR T cells may interact with costimulatory receptor ligand‐expressing APCs within the tumor microenvironment.

Bi‐ or multicistronic viral vectors allow delivery of additional genes alongside the CAR, presenting alternative options to provide CAR T cell costimulation. For example, Curran *et al*.^64^ reported that second‐generation CD28‐costimulated anti‐CD19 CAR T cells exhibited improved cytotoxicity against B‐cell lymphoma in xenograft models when the CAR was delivered alongside a copy of the CD40L gene using a bicistronic vector. Similarly, Zhao *et al*.^27^ found that delivering 4‐1BBL alongside a CD28‐costimulated second‐generation CAR improved antitumor activity. By compartmentalising stimulatory signals (CD3ζ and CD28) onto two separate CARs directed against different antigens, Wilkie *et al*.^65^ were able to generate CAR T cells that required target cell co‐expression of each target antigen for effective tumor lysis. Meanwhile, CAR T cells capable of inducible expression of a synthetic costimulatory protein incorporating MyD88 and CD40 signalling domains (Figure 3) have entered clinical trials as a prostate stem cell antigen‐specific CAR T cell BPX‐601, with early reports of clinical responses.^17^

The potency of the combination of MyD88 and CD40 endodomains might be explained by the non‐redundant signalling induced by this signalling pair. For example, MyD88, but not CD40, greatly enhanced IL‐6 production in T cells, a feature which might contribute to enhanced central memory cell formation and anti‐cancer activity, but might also increase the risk of CRS. Similar to CD28‐mediated signalling, ligation of T cell‐expressed CD40 induces transcription factor activation and cytokine production, but with greater NF‐κB activation.^66^ Unlike CD28, CD40‐mediated costimulation is able to recruit TRAF to membrane rafts.^66^

A further method of recruiting costimulatory signals is to generate CAR T cells from T cells expressing a TCR of defined specificity, such as those directed against a virus. In this case, stimulation of the native TCR on CAR T cells by viral antigens might provide additional signals 1 and 2, the latter through the action of microbial pathogen‐associated molecular patterns on APCs*,* enhancing activity against the target of the CAR or increasing persistence. Rossig *et al*. reported that CAR T cells generated from EBV‐specific T cells had superior persistence to those prepared from bulk CAR T cells. Moreover, persistence of CAR T cells expressing EBV‐specific TCRs was enhanced by EBV‐directed vaccination.^67^

## Optimal use of costimulatory domains

### Disease‐specific indications for use of costimulatory domains

While there is redundancy in costimulatory molecule signalling pathways, different costimulatory domains within CARs can induce different downstream signalling events and favor induction of distinct T cell phenotypic and functional characteristics. For example, costimulatory molecules appear to differ in their ability to induce CAR T cell differentiation to effector, memory or cytokine‐polarised subsets.^51^, ^68^

**Figure 4:**
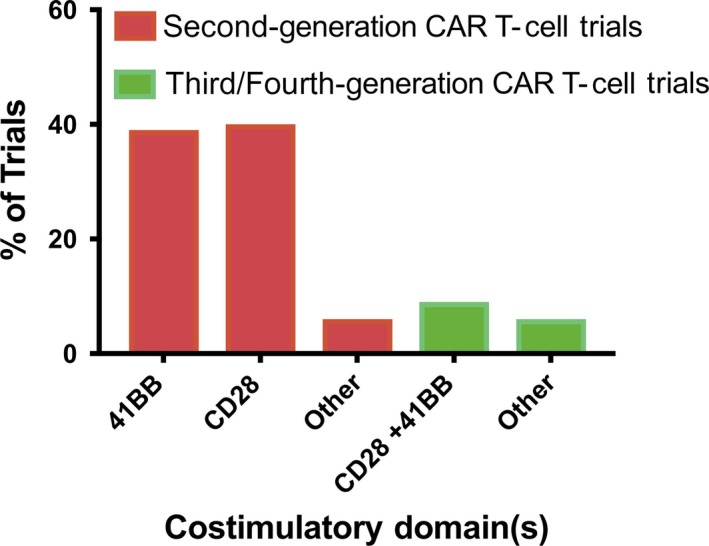
Survey of registered clinical trials utilising distinct costimulator domain structures. A search for anti‐CD19 chimeric antigen receptor (CAR) T cell trials registered at ClinicalTrials.gov obtained information on costimulatory domains in 55 trials. Information on costimulatory domains in a further 15 trials was obtained by emailing the trial contact on ClinicalTrials.gov, leading to a total of 69 anti‐CD19 CAR T cell trials available for data analysis. The second‐generation CAR T cell trials in ‘other’ include 2 trials where an admix of second‐generation CARs containing 41BB and CD28 was administered, and two trials where either 41BB or CD28 containing second‐generation CARs were administered. The third/fourth‐generation CAR T cell trials in ‘other’ includes one trial where an admix of a third‐generation CAR utilising CD28 and 41BB costimulation plus a CD28 containing second‐generation CAR was administered, one trial where costimulation of CD28 and TLR2 was utilised, one trial where costimulation of CD28 and CD27 was utilised and one trial where costimulation of 41BB and CD27 was utilised.

The most frequently employed costimulatory domains, CD28 and 4‐1BB, are each incorporated in licensed second‐generation anti‐CD19 CAR T cell therapies (see Figures 3 and 4). These products are administered at similar doses, with broadly similar efficacy and safety profiles. However, preclinical models and exploratory comparisons between single‐arm clinical studies suggest CAR T cells incorporating each of these domains may differ qualitatively.^11^, ^24^, ^25^, ^29^, ^50^, ^56^, ^68^

In animal studies, CD28‐costimulated CAR T cells exhibit improved early expansion and cytotoxic activity compared to their 41BB‐costimulated counterparts *in vivo*, while 41BB‐costimulated CAR T cells exhibit better long‐term persistence.^27^ Consistent with this, pharmacokinetic analyses of phase 2 CAR T cell clinical trials in r/r B‐NHL suggest that CD28‐containing second‐generation anti‐CD19 CAR T cells peak earlier but persist less well than 41BB‐incorporating CAR T cells.^30^, ^36^

The optimal costimulatory domain might depend upon clinical indication. With the proviso that comparison between trials is complicated by differences in trial populations, study design and the CAR T cell manufacturing process, results of the largest single‐arm studies suggest that in aggressive B‐NHL, a malignancy of mature B cells, CD28‐costimulated CAR T cells are associated with numerically higher response rates than 4‐1BB CAR T cells,^30^, ^36^ and that durable remissions are observed despite apparent loss of CAR T cell persistence.^69^ In contrast, in B‐ALL, a malignancy of precursor B cells, 41BB‐costimulated CAR T cells may elicit numerically longer median progression‐free survival rates,^38^, ^70^ and a loss of CAR T cell persistence often heralds relapse.^37^ This raises the possibility that optimal responses in aggressive B‐NHL may require robust early cytotoxic activity, as provided by CD28‐costimulated CAR T cells, while optimal responses in B‐ALL may require longer term persistence, as provided by 41BB‐costimulated CAR T cells.

Ultimately, randomised clinical trials prospectively comparing CAR T cells incorporating various costimulatory domains are needed; such trials comparing second‐generation CD28 and 4‐1BB CAR T cells are underway and will shed light on the impact of costimulatory domain selection (ClinicalTrials.gov numbers NCT03191773 and NCT03076437, see Figure 4).

### Position within the CAR construct

Positioning of costimulatory domains within the endodomain of a CAR can influence CAR T cell activity. A CD28‐CD3ζ CAR, with the CD28 domain in the membrane‐proximal position, performed better in terms of T cell proliferation and IL‐2 production than a CD3ζ‐28 CAR.^12^, ^13^ Incorporation of both the transmembrane and intracellular domains of the CD28 domain within CD28‐CD3ζ was required for optimal surface expression of the CARs.^12^

Guedan *et al*. provide additional insight into the effect of positioning of intracellular costimulatory domains within CARs. Combining 4‐1BB and ICOS costimulatory domains, they observe that the domain proximal to the cell membrane has a dominant effect on cytokine profile, compared to the more distal domain: an ICOS‐41BB‐CD3ζ construct resulted in a CAR T cell cytokine profile similar to that of an ICOS‐CD3ζ construct (with greater IL‐17 production), while a 41BB‐ICOS‐CD3ζ construct elicited a profile similar to that of 41BB‐CD3ζ (with greater IL‐6 and IL‐13).^68^

Pule *et al*.^49^ showed expression of a third‐generation CD28‐OX40‐CD3ζ CAR led to 10‐fold and fivefold more T cell IL‐2 and TNF‐α production, respectively, yet the same level of IL‐10, compared with a CD28‐CD3ζ construct. In contrast, Hombach *et al*.^48^ found that a CD28‐CD3ζ‐OX40 construct led to reduced IL‐10 production in comparison with a CD28‐CD3ζ CAR and that IL‐2 production was also lower.

Taken together, these studies suggest that positioning of costimulatory domains within CARs can influence CAR T cell function.

## Limitations of CAR costimulation

### Tonic signalling and CAR T cell exhaustion

Costimulatory domains may have negative, as well as positive, impacts on CAR T cell function. Clustering of CAR scFvs independently of antigen could cause tonic signalling within CAR T cells, leading to exhaustion or AICD. Costimulatory domains within CARs may contribute to this phenomenon. For example, Long *et al*.^71^ described exhaustion of CAR T cells because of tonic signalling in T cells expressing anti‐GD2 CARs incorporating a CD28, but not a 4‐1BB, costimulatory domain. These effects appear to be scFv‐, and even vector‐, dependent: anti‐CD19 CARs incorporating the same CD28 costimulatory domain were not susceptible to exhaustion,^71^ while Gomes‐Silva *et al*.^72^ described exhaustion because of tonic signalling in response to 41BB‐costimulated anti‐CD19 CAR T cells, attributing this to positive feedback on the gammaretroviral‐derived long terminal repeat‐based transgene promoter.

### Safety considerations

Toxicities following CAR T cell therapy present a major limitation of CAR T cell therapies. Of particular concern are CRS and neurotoxicity (CRES or ICANS).^11^, ^29^, ^59^ Both CRS and neurotoxicity are a consequence of cytokine production by activated CAR T cells, which in turn leads to activation of host myeloid cells to amplify cytokine production and enhance inflammation via IL‐1 and IL‐6.^11^, ^29^, ^59^

Direct comparisons of CRS and neurotoxicity rates between CAR T cell trials utilising different costimulatory domains are complicated by differing baseline characteristics, disease burdens, toxicity grading systems and toxicity management strategies.^73^ For example, rates of severe (grade ≥ 3) CRS and neurotoxicity among recipients of the CD28‐costimulated anti‐CD19 CAR T cell product axicabtagene ciloleucel were 11% and 28%, in comparison with 24% and 16%, respectively, among recipients of the 41BB‐costimulated product tisagenlecleucel for a similar indication (B‐NHL).^30^, ^36^ While these rates appear similar, the former study used the Lee *et al*. grading system for CRS, while the latter used the University of Pennsylvania (UPenn) grading scale.^73^, ^74^ CRS requiring ‘low‐dose’ vasopressors may be graded at grade 2 using the Lee *et al*. scale, but grade 3 using the UPenn scale.^73^ Moreover, increasing clinical experience in the early recognition and management of CRS and neurotoxicity is likely to reduce the incidence of severe toxicities, the early introduction of anti‐IL‐6 therapy and corticosteroids being associated with lower rates of life‐threatening complications.^74^

Both CRS and neurotoxicity are typically early events after CAR T cell administration. CRS in particular often occurs during or near the peak of CAR T cell expansion. The greater early expansion of, and cytokine production by, CD28‐costimulated CAR T cells^27^ raises the possibility that this domain might be associated with higher CRS rates. However, no direct comparisons have yet been reported, and fatal CRS and neurotoxicity events been reported in trials of CAR T cells utilising both 41BB and CD28 costimulatory domains.^30^, ^70^

By combining costimulatory domains to elicit greater cytokine production, third‐generation CAR T cells might plausibility exacerbate toxicity. Early‐phase clinical trials of third‐generation anti‐CD19 CAR T cells suggest rates of severe CRS and neurotoxicity are 12–33%, similar to those reported following treatment with second‐generation products.^53^, ^56^, ^58^ However, the number of recipients of third‐generation CAR T cells in the published literature is low, and the results of additional trials, including some currently recruiting studies, will be needed to accurately assess toxicity risk (see Figure 4).

While the contribution of costimulatory domains to toxicity is not yet clear, many studies indicate that a high disease burden, particularly within the bone marrow, correlates with severe CRS and neurotoxicity risk.^29^, ^70^ Patient selection and early CRS and neurotoxicity recognition are critical. Ultimately, only prospective randomised trials comparing the efficacy and safety of CARs expressing different costimulatory domains will reliably determine the optimal constructs for each therapeutic indication.

## Future perspectives

The incorporation of various intracellular signalling domains within CARs enables an unprecedented degree of control over T cell activity and fate. Future CAR designs may benefit from the discovery of new costimulatory pathways, as well as the exploitation of known accessory molecules. The optimal use of these new CARs will require an increased understanding of the impact of CAR costimulatory domains across the spectrum of cancer subtypes. For example, while the CD28 domain is effective for the treatment of subtypes of B‐NHL, 41BB‐based CARs may be more effective for the longer term CAR T cell persistence required to eradicate residual disease in B‐ALL. Current costimulatory domains within CARs are direct replicates of natural costimulatory domains; however, synthetic costimulatory domains offer the possibility to combine distinct signal pathways, while avoiding possible interactions between costimulatory domains and endogenous inhibitory pathways. Incorporation of new costimulatory domains to maximise CAR T cell expansion and persistence will have to take into account the possible increased risk of CRS and neurotoxicity. Clinician‐inducible, or auto‐inducible, control systems offer new strategies to mitigate such risks. For example, the use of small molecule inhibitors to control costimulatory activity *in vivo*,^17^ or synthetic sensing and gene regulation systems^75^ to modulate CAR T cell activity might prove useful adjuncts. Further comparisons of CARs both *in vitro* and *in vivo* will inform rational selection of costimulatory domains for assessment in novel constructs for clinical indications. For early clinical development, trial designs comparing two CAR T cells simultaneously administered to the same patient have provided invaluable evidence of the pharmacokinetic effect of costimulatory domains,^25^, ^28^, ^56^ and could provide further information about the impact of the selection and sequence of costimulatory domains within CAR T cells. Ultimately, widespread adoption of the most effective and safe CAR T cell therapies will require large‐scale clinical trials comparing lead constructs.
